# Coexistence of pulmonary arterial hypertension and straight back syndrome in a patient with a novel BMPR2 variant affecting cytoplasmic tail domain

**DOI:** 10.1186/s40001-024-01810-x

**Published:** 2024-04-01

**Authors:** Mi Tang, Jun Luo, Qingqing Liu, Jie Song

**Affiliations:** 1 Department of Cardiovascular Surgery, The Second Xiangya Hospital, Central South University, No. 139 Ren-Min Road, Changsha, 410011 China; 2 Department of Cardiovascular medicine, The Second Xiangya Hospital, Central South University, No. 139 Ren-Min Road, Changsha, 410011 China; 3 Department of Respiratory and Critical Care, The Second Xiangya Hospital, Central South University, No. 139 Ren-Min Road, Changsha, 410011 China

**Keywords:** Pulmonary arterial hypertension, Straight back syndrome, BMPR2, Genetic variation, Cytoplasmic tail

## Abstract

**Background:**

Pathologic variants in the bone morphogenetic protein receptor-2 (*BMPR2*) gene cause a pulmonary arterial hypertension phenotype in an autosomal-dominant pattern with incomplete penetrance. Straight back syndrome is one of the causes of pseudo-heart diseases. To date, no cases of idiopathic or heritable pulmonary arterial hypertension with straight back syndrome have been reported.

**Case presentation:**

A 30-year-old female was diagnosed with pulmonary arterial hypertension by right heart catheterization. Computed tomography revealed a decreased anteroposterior thoracic space with heart compression, indicating a straight back syndrome. Genetic analysis by whole exome sequencing identified a novel c.2423_2424delGT (p.G808Gfs*4) germline frameshift variant within *BMPR2* affecting the cytoplasmic tail domain.

**Conclusions:**

This is the first report of different straight back characteristics in heritable pulmonary arterial hypertension with a novel germline *BMPR2* variant. This finding may provide a new perspective on the variable penetrance of the pulmonary arterial hypertension phenotype.

**Supplementary Information:**

The online version contains supplementary material available at 10.1186/s40001-024-01810-x.

## Introduction

Pulmonary arterial hypertension (PAH), referring to group 1 pulmonary hypertension, is characterized by an unreversible increase in pulmonary vessel resistance (PVR) leading to right heart failure [1]. The prevalence of PAH ranges from 48 to 55 cases per million adults in recent registries [2]. Genetic heterogeneity, predominantly due to mutation in the bone morphogenetic protein receptor-2 (*BMPR2*) gene, is one of the most important associated factors for the etiologies of the disease [3]. Approximately 85% of families with documented heritable PAH (HPAH) in one or more members carry a mutation in the *BMPR2* gene. In sporadic PAH cases, 14–35% of patients, mostly in idiopathic PAH (IPAH), have an identified underlying pathogenic variant in *BMPR2* [3]. The structural change of PAH is demonstrated as an enlarged right heart with reduced function resulting from an irreversible elevation of PVR. Alterations of heart and great vessels’ compression may occur in individuals with straight back syndrome due to an absence of normal physiological curvature of the dorsal spine [4]. Here, we present a rare case of a PAH patient associated with straight back syndrome and surprisingly identified a novel variant locus in the *BMPR2* gene.

## Case presentation

A 30-year-old female was referred to our hospital in 2020 with a main complaint of intermittent dyspnea for five years, aggravated with syncope and exertional dyspnea for 6 months and exhibited a World Health Organization functional class (WHO-FC) III status. She was diagnosed with primary PAH at age 25 in 2016 in another hospital by right heart catheterization with a mean pulmonary arterial pressure (mPAP) of 43 mmHg and a PVR of 12.5 Wood units. The patient has no family history of pulmonary or cardiac vascular disease, and no orthopedic problem. Her BMI was 19.04 kg/m^2^. A physical examination revealed a blood pressure of 109/77 mmHg, a pulse rate of 77 beats/min, a respiratory rate of 20 breaths/min, a pulse oximetry of 94% on room air and a body temperature of 36.1 ℃. Cardiopulmonary examination revealed clear lung fields and an accentuated pulmonic second heart sound.

**Fig. 1:**
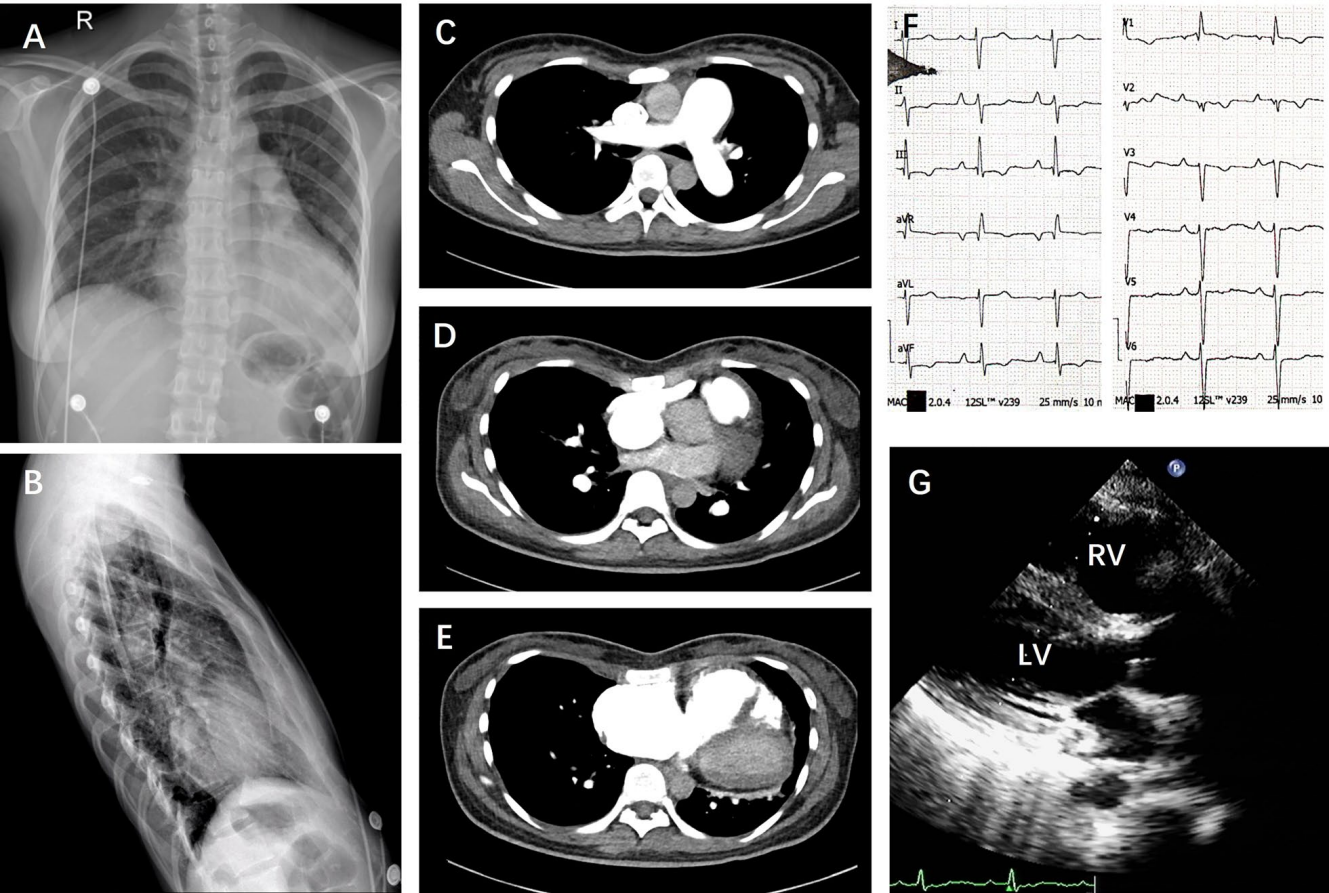
Chest X-ray and CT scans of the patient. **A** Posteroanterior chest X-ray showing accentuation of vessels, prominence of pulmonary artery trunk and an enlarged heart shadow. **B** Lateral chest X-ray showing a straight curvature of the thoracic spine. **C** CT scan showing a dilated pulmonary artery and a narrow anteroposterior interval. **D** CT scan showing the heart is compressed in a narrow thoracic space with the right atrium adjacent to the posterior of the sternum. **E** CT scan showing a dilated right ventricle. **F** Electrocardiogram demonstrating a sinus rhythm with right axis deviation, and inverted T waves in lead II, III, aVF, V1–V4. **G** Echocardiogram showing of a dilated right ventricle and minimal pericardial effusion

Chest radiography showed prominent hilar pulmonary arteries, with a cardiothoracic ratio of 0.66 (Fig. 1A). Notably, the enlarged heart was compressed in a narrow anteroposterior thoracic space due to the straightening of the dorsal spine in the lateral view (Fig. 1B). Computed tomography (Fig. 1C–E) confirmed an enlargement of the right heart with a dilated main pulmonary artery of 30 mm in diameter. The ratio of the anteroposterior to the transverse chest diameter is 0.25, which is much smaller than normal.

**Fig. 2:**
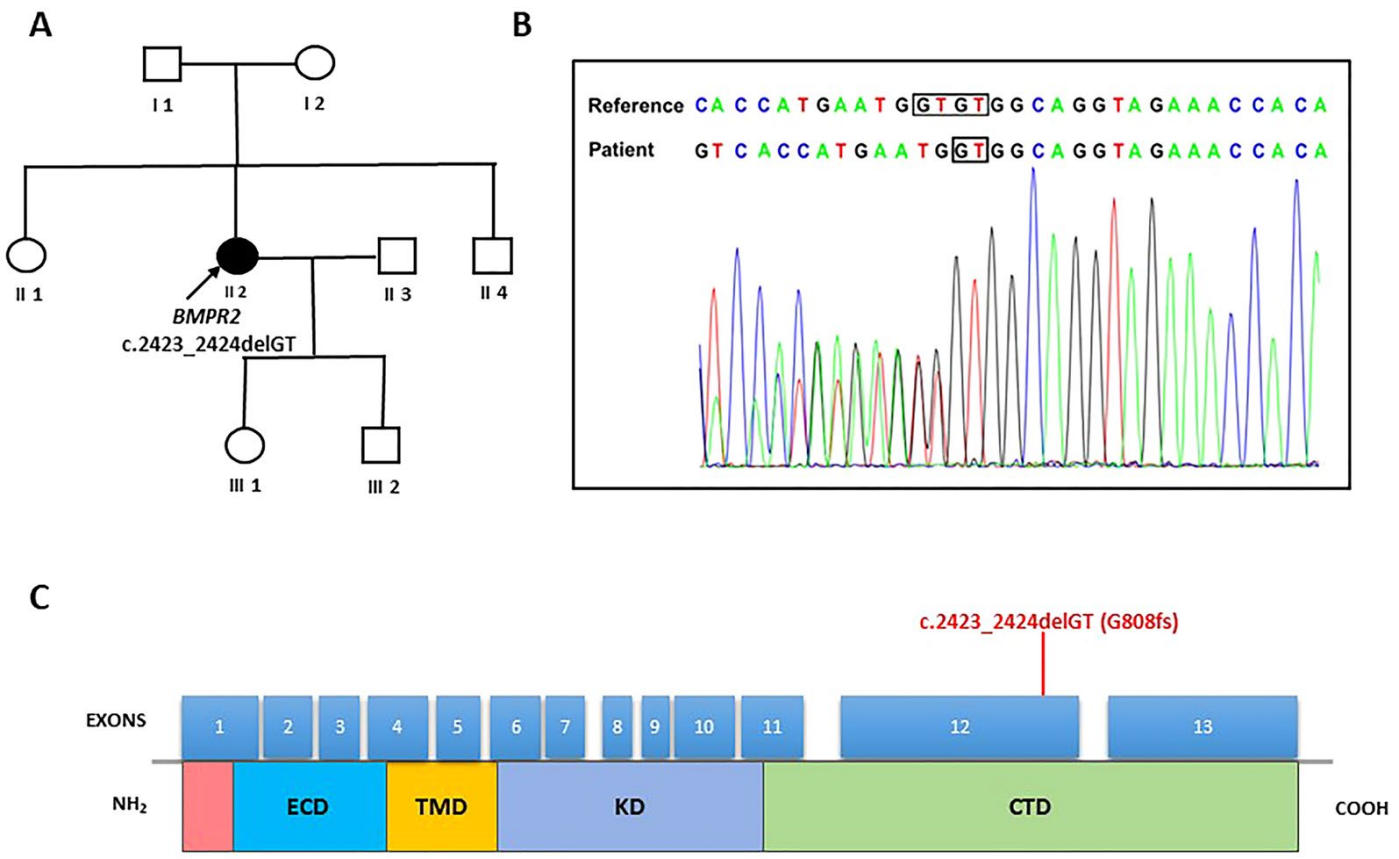
Identification of the novel *BMPR2* variant. **A** Family pedigree of the patient with PAH with straight back syndrome. The filled symbol represents the affected patients, empty circles/squares represent the unaffected members. The index patient (II 2) is indicated by a black arrow. **B** and **C** Sanger sequencing showing the variant of c.2423_2424delGT in the 12th exon within *BMPR2*, which is located in the cytoplasmic tail domain. ECD, extracellular domain; TMD, transmembrane domain; KD, kinase domain; CTD, cytoplasmic tail domain

An electrocardiogram disclosed signs of right axis deviation, right atrial and right ventricular enlargement (Fig. 1F). Transthoracic echocardiography showed that the right heart was dilated and the left ventricle was compressed (Fig. 1G). The tricuspid annular plane systolic excursion (TAPSE) was measured at only 12 mm and the right ventricular fractional area change (RV-FAC) was 26%, reflecting a reduced right heart systolic function. Right heart catheterization (Table 1) revealed severe precapillary pulmonary hypertension, with an elevated mPAP of 50 mmHg, pulmonary capillary wedge pressure (PAWP) of 8 mmHg, and PVR of 14.29 Wood units. An acute vasoreactivity testing was performed to show a negative response. Pulmonary artery angiogram indicated no evidence of pulmonary embolism or arteriovenous malformations. Blood tests revealed elevated levels of total bilirubin (38.9 µmol/l), direct bilirubin (11.4 µmol/l), and N-terminal pro-brain natriuretic peptide (NT-proBNP, 3020 pg/ml). Laboratory tests for autoimmune diseases and human immunodeficiency virus were all negative. The patient had no detectable associated medical agents known to cause PAH. Therefore, a genetic analysis by whole exome sequencing on a next generation sequencing approach was performed. A novel heterozygous frameshift germline variant in the exon 12 of the *BMPR2* gene (c.2423_2424delGT, p.G808Gfs*4, NM_001204) was identified and subsequently validated (Fig. 2) by Sanger sequencing. The variant was absent in the Exome Sequencing Project, 1000 Genomes Project, or Exome Aggregation Consortium. This is a previously unreported variant, predicted to result in deleterious functional consequences. The deletion of two bases causes a frameshift alteration, which is defined as a null variant by the ACMG Standards and Guidelines [5], predicted premature protein truncation through nonsense-mediated decay. Hence, the patient was diagnosed with HPAH and straight back syndrome.

**Table 1:** Clinical parameters

Date	2016.03	2020.07	2023.03
Age	25	30	32
Right heart catheterization			
PAP (S/D/M, mmHg)	65/29/43	87/29/50	–
PAWP (mmHg)	8	8	–
RAP (mmHg)	10	10	–
CI (L/min/m^2^)	2.08	2.16	–
PVR (Wood Units)	12.5	14.29	–
SVR (Wood Units)	27.38	23.8	–
SaO_2_ (%)	94	93	–
Echocardiogram			
LVEDd (mm)	38	39	36
LVEF (%)	59	50	52
RVd (mm)	35	41	42
PA (mm)	32	32	33
Peak TRV (m/s)	3.2	2.8	3.1
TAPSE (mm)	–	12	15
RV-FAC (%)	–	26	29
IVC (mm)	12	15	13
Pericardial effusion	No	Minimal	No
NT-proBNP (pg/ml)	628	3020	352
6MWD (m)	496	294	482
WHO-FC	II	III	II
Medication	Silenafil, Bosentan	Sildenafil, Macitentan, Treprostinil	Sildenafil, Macitentan, Selexipag

PAP: pulmonary arterial pressure, S: systolic, D: diastolic, M: mean, PAWP: pulmonary arterial wedge pressure, RAP: right arterial pressure, CI: cardiac index, PVR: pulmonary vascular resistance, SVR: systemic vascular resistance, SaO_2_: arterial oxygen saturation, LVEDd: left ventricular end diastolic diameter, LVEF: left ventricular ejection fraction, RVd: right ventricle diameter, PA: pulmonary artery, TRV: tricuspid regurgitation velocity, TAPSE: tricuspid annular plane systolic excursion, RV-FAC: right ventricular fractional area change, IVC: inferior vena cava, NT-proBNP: N-terminal pro-brain natriuretic peptide, 6MWD: 6-min walking distance, WHO-FC: World Health Organization functional class

The patient was administrated sildenafil (25 mg three times daily) and bosentan (125 mg twice daily) therapy since 2016. As her symptoms worsened, the level of NT-proBNP was 3020 pg/ml with the dyspnea evaluated to be a WHO-FC III status on admission. Disease severity was evaluated as intermediate–high risk during this visit. The subsequent targeted therapy was escalated to a combination of sildenafil (25 mg three times daily), macitentan (10 mg once daily), and treprostinil injection (a gradual up-titration to 20 ng/kg/min). After six months, her heart function was improved to WHO-FC II with an NT-proBNP level reduced to 352 pg/ml. Given her clinical improvement, a plan was made to transition from treprostinil injection to oral Selexipag up-titrated to 1.0 mg twice daily. No follow-up hemodynamic evaluation has been performed to date. The TAPSE was increased to 15 mm and the RV-FAC was of 29% by echocardiogram during the last follow-up on March 2023 (Table 1). Her heart function remained to be WHO-FC II. The patient is currently comfortable with normal activities with no signs of symptomatically worsening, showing her benefits from triple combination therapy.

## Conclusions and discussion

This is a rare case of HPAH with *BMPR2* variant coexisting with straight back syndrome. *BMPR2* encodes a type II receptor of the TGF-β family of signaling molecules, which participates in a multitude of cellular processes including pulmonary artery endothelial barrier function [6], smooth muscle cell proliferation, apoptosis and migration [7]. Since the first *BMPR2* mutation was identified in 2000, the use of advanced sequencing has allowed for the identification of more than ten validated disease-causing genes in PAH, mostly linked to the transforming growth factor beta (TGF-β)/SMAD pathway [8]. However, not all *BMPR2* mutation carriers will develop the disease during their lifetime due to a reduced penetrance. The penetrance of *BMPR2* mutations is age-dependent and has been estimated to be around 42% in women and 14% in men at most [9]. Previous studies have reported that HPAH patients harboring *BMPR2* mutations have an earlier onset of diagnosis and a severe disease prognosis with a short life span [10]. The variant in this case is in the 12th exon of the *BMPR2* gene, located in the cytoplasmic tail domain of the polypeptide (Fig. 2C and Additional file 1: Fig. S1A). According to the ACMG Standards and Guidelines [5], the variant is classified as pathogenic (Ib) based on evidence PVS1 (null variant in *BMPR2*), PM2 (absent from controls), and PM6 (assumed de novo, but without confirmation of paternity and maternity). Up to date, over 800 PAH-associated variants within *BMPR2* have been reported, including more than 50% of all pathologic variants in the kinase domain and approximately 20% of them in the extended cytoplasmic tail domain (Additional file 1: Fig. S1B). Functional studies confirmed the loss of function in the BMP/SMAD pathway resulting from mutations affecting the kinase domain of *BMPR2* [11]. However, the effect of mutations in the cytoplasmic tail was able to retain most of its functions in SMAD signaling [12]. Compared with the kinase domain, mutations in the cytoplasmic tail cause a milder phenotype with later disease onset and milder hemodynamic compromise at diagnosis, which is more similar to the disease profile in IPAH [11]. In this case, the patient with a cytoplasmic tail *BMPR2* variant may have a milder phenotype other than manifested with a relatively earlier onset at age 25. This indicates that additional modifying factors such as variants in untranslated regions or non-genetic factors might cause disease manifestation together, which is also called as “second-hit” model hypothesis in our previous report [13].

Straight back syndrome is a skeletal deformity by the loss of normal thoracic kyphosis. Since lacking the normal degree of dorsal curvature, the spine becomes vertical and a narrow space between the spine and the sternum which can be seen on the lateral chest X-ray [4]. In this case, the ratio of anteroposterior to transthoracic diameter was 0.25, which was compatible with the criteria of straight back syndrome with a ratio of 1/3 or less [4, 14]. It occurs in thin individuals and is usually asymptomatic. Due to the proximity of the right heart to the anterior wall resulting from the forward and leftward shift of the right heart and main pulmonary artery, some have a cardiac murmur or an increased amplitude of the pulmonic sound resembling an atrial septal defect or other organic heart diseases [4]. In this patient, the increase in the intensity of the pulmonic sound might be caused by both dilation and proximity of the main pulmonary artery. In a previous report, the absence of normal dorsal curvature of the spine was present in 9.2% of congenital heart disease [15]. In addition, a reduced anteroposterior dimension of the thorax may cause asynchronous papillary muscle motion linked to mitral valve prolapse [16]. The patient in this case was diagnosed with PAH, ruling out congenital heart defect, valve disorder or other vessel malformation screened by echocardiogram and pulmonary artery angiogram. Since the straight thoracic spine has not been previously reported in patients coexisting with IPAH or HPAH, so far it is not clear whether this skeletal abnormality has any correlation with the phenotypic features of PAH. However, the decreased sagittal diameter of the thoracic cage can result in a compressed heart, which might subsequently strengthen the load of pulmonary circulation. Therefore, this alteration may serve as an additional modifier to increase the penetrance of PAH disease manifestation in this *BMPR2* variant carrier.

To our knowledge, this is the first report of a new variant in the cytoplasmic tail domain within *BMPR2* leading to HPAH coexisting with a straight back syndrome, providing an explanation for the possible PAH phenotype manifestation. Our observation widens the *BMPR2* variant landscape and strengthens the importance of a correct diagnosis of PAH. The diagnostic workflow with the molecular test should be more widely applied to allow for an earlier precision diagnosis and tailored interventions to delay the onset or treat PAH in the future.

## Methods

### Subjects and clinical characterization

Clinical data were collected including echocardiogram, X-ray computed tomography angiography, right heart catheterization and blood test.

### DNA isolation

The EDTA-treated blood was collected from the peripheral blood of the patient. Genomic DNA was extracted using the Blood Genome Column Medium Extraction Kit (Kangweishiji, China), according to the manufacturer’s recommendation.

### Whole exome sequencing

The xGen Exome Research Panel v1.0 (IDT, Iowa, USA) includes 429,826 individually synthesized and quality-controlled probes, targeting 39 Mb coding region (19,396 genes) of the human genome and covers 51 Mb of end-to-end tiled probe space. High-throughput sequencing was performed on the Illumina NovaSeq 6000 series sequencer (Illumina, San Diego, CA, USA). The sequencing process was performed by Chigene Translational Medicine Research Center (Beijing. China). More than 99% of targeted sequences were sequenced. Raw data were processed by Fastp software for adapters removing and filtering out low-quality reads (Q score of below 20). Sequencing reads passing quality filters were aligned to the Ensemble GRCh37/hg19 reference genome using Burrows–Wheeler Aligner software. Single Nucleotide Polymorphisms (SNPs), short Indel calling and base quality score recalibration were conducted by Genome Analysis Toolkit software. After screening SNPs and Indels according to sequencing depth and high quality, reliable variants were obtained.

### Variant analysis

The online system independently developed by Chigene (www.chigene.org) was used to annotate database-based minor allele frequencies (MAFs). The MAF < 0.1% in public databases (1,000 genomes, dbSNP, ESP, and ExAC database) was considered; Missense variants were analyzed by in silico tools (Provean, Sift, Polypen2, Mutation Taster, M-Cap, and Revel) to predict protein product structure variation. As a prioritized pathogenicity annotation to ACMG guidelines, OMIM, HGMD and ClinVar databases were used as references of pathogenicity of every variant.

### Sanger sequencing

The identified variant c.2423_2424delGT (p.G808Gfs*4) was further validated by Sanger sequencing using ABI 3730 sequencer (Applied Biosystems, Foster City, CA, USA). The origin of this newly identified variant might have been explored through the parents’ DNA while the samples were not available. The primer sequences were designed as follows: Forward: 5′-CCGGCTAAAATTTGGCAGCA-3′, Reverse: 5′-CCAGCTTGTTGCTCTCGTCT-3′.

### Supplementary Information

**Additional file 1: Fig. S1.** Overview of the *BMPR2* variations and protein domains.

## Data Availability

The data set supporting the conclusions of this article is available in the GSA for Hunam repository. The accession number can be found below: GSA for Hunam (https://ngdc.cncb.ac.cn/gsa-human/), No. HRA005110. Further inquiries are available from the corresponding author on reasonable request.

## Abbreviations

PAH: Pulmonary arterial hypertension
PVR: Pulmonary vessel resistance
BMPR2: Bone morphogenetic protein receptor-2
HPAH: Heritable PAH
IPAH: Idiopathic PAH
WHO-FC: World Health Organization functional class
mPAP: Mean pulmonary arterial pressure
TAPSE: Tricuspid annular plane systolic excursion
RV-FAC: Right ventricular fractional area change
PAWP: Pulmonary capillary wedge pressure
NT-proBNP: N-terminal pro-brain natriuretic peptide
TGF-β: Transforming growth factor beta

## References

[1] Humbert M, Kovacs G, Hoeper MM (2022). 2022 ESC/ERS Guidelines for the diagnosis and treatment of pulmonary hypertension. Eur Heart J.

[2] Leber L, Beaudet A, Muller A (2021). Epidemiology of pulmonary arterial hypertension and chronic thromboembolic pulmonary hypertension: identification of the most accurate estimates from a systematic literature review. Pulm Circ.

[3] Machado RD, Southgate L, Eichstaedt CA (2015). Pulmonary arterial hypertension: a current perspective on established and emerging molecular genetic defects. Hum Mutat.

[4] De Leon AC, Perloff JK, Twigg H (1965). The straight back syndrome: clinical cardiovascular manifestations. Circulation.

[5] Richards S, Aziz N, Bale S (2015). Standards and guidelines for the interpretation of sequence variants: a joint consensus recommendation of the American college of medical genetics and genomics and the association for molecular pathology. Genet Med.

[6] Hwangbo C, Lee H-W, Kang H (2017). Modulation of endothelial bone morphogenetic protein receptor type 2 activity by vascular endothelial growth factor receptor 3 in pulmonary arterial hypertension. Circulation.

[7] Humbert M, Guignabert C, Bonnet S (2019). Pathology and pathobiology of pulmonary hypertension: state of the art and research perspectives. Eur Respir J.

[8] Morrell NW, Aldred MA, Chung WK (2019). Genetics and genomics of pulmonary arterial hypertension. Eur Respir J.

[9] Larkin EK, Newman JH, Austin ED (2012). Longitudinal analysis casts doubt on the presence of genetic anticipation in heritable pulmonary arterial hypertension. Am J Respir Crit Care Med.

[10] Evans JD, Girerd B, Montani D (2016). BMPR2 mutations and survival in pulmonary arterial hypertension: an individual participant data meta-analysis. Lancet Respir Med.

[11] Girerd B, Coulet F, Jais X (2015). Characteristics of pulmonary arterial hypertension in affected carriers of a mutation located in the cytoplasmic tail of bone morphogenetic protein receptor type 2. Chest.

[12] Nohe A, Hassel S, Ehrlich M (2002). The mode of bone morphogenetic protein (BMP) receptor oligomerization determines different BMP-2 signaling pathways. J Biol Chem.

[13] Eichstaedt CA, Song J, Benjamin N (2016). EIF2AK4 mutation as “second hit” in hereditary pulmonary arterial hypertension. Respir Res.

[14] Kambe M (2006). Straight back syndrome and respiratory failure. Jpn Med Assoc J.

[15] Gooch AS, Maranhao V, Goldberg H (1967). The straight thoracic spine in cardiac diagnosis. Am Heart J.

[16] Schiavone WA (2021). Straight back syndrome as a clue to diagnosing asymptomatic congenital valvular heart disease and limiting the risk of weightlifting. J Osteopath Med.

