# Supplementary material for: Coexistence of pulmonary arterial hypertension and straight back syndrome in a patient with a novel BMPR2 variant affecting cytoplasmic tail domain
Source: Eur J Med Res. 2024 Apr 1;29:209. doi: 10.1186/s40001-024-01810-x (PMC10983711; doi:10.1186/s40001-024-01810-x)
Supplement: Supplementary file 1 — Additional file 1: Fig. S1. Overview of the BMPR2 variations and protein domains. [file 40001_2024_1810_MOESM1_ESM.docx]

Additional File 1 Overview of the BMPR2 variations and protein domains.


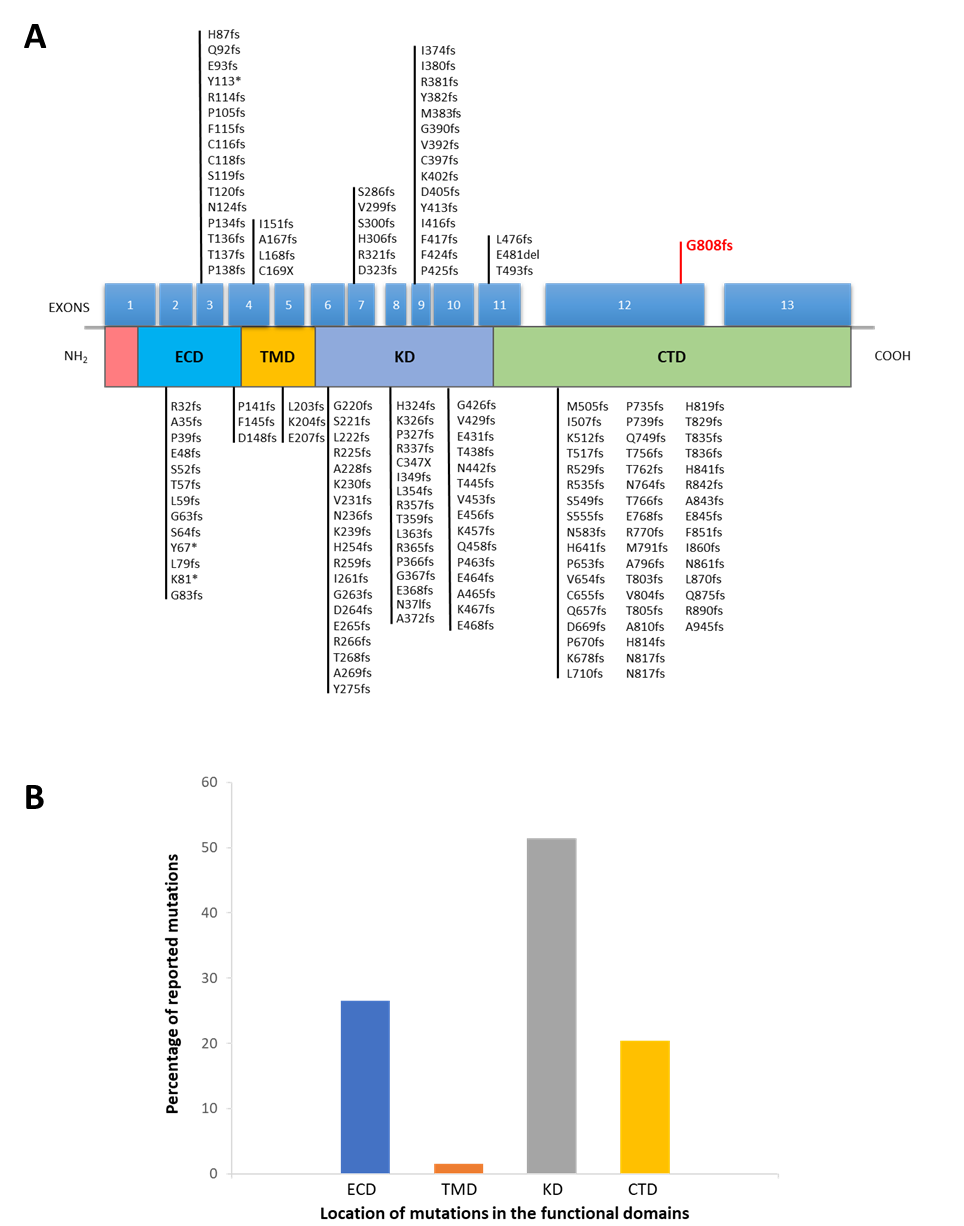


**Fig. S1** Overview of the *BMPR2* variations and protein domains. **A** Overview of reported small insertions and deletions across the *BMPR2* gene [1-13]. The red font symbolizes the newly identified variant within the cytoplasmic tail domain. **B** The proportion of all distinct *BMPR2* mutations in different functional domains. ECD, extracellular domain; TMD, transmembrane domain; KD, kinase domain; CTD, cytoplasmic tail domain.

# References

1. Zhu N, Gonzaga-Jauregui C, Welch CL et al: Exome Sequencing in Children With Pulmonary Arterial Hypertension Demonstrates Differences Compared With Adults. Circulation: Genomic and Precision Medicine 2018, 11(4).

2. Yang H, Zeng Q, Ma Y et al: Genetic analyses in a cohort of 191 pulmonary arterial hypertension patients. Respiratory Research 2018, 19(1).

3. van der Bruggen CE, Happé CM, Dorfmüller P et al: Bone Morphogenetic Protein Receptor Type 2 Mutation in Pulmonary Arterial Hypertension. Circulation 2016, 133(18):1747-60.

4. Sztrymf B, Coulet F, Girerd B et al: Clinical Outcomes of Pulmonary Arterial Hypertension in Carriers of BMPR2 Mutation. American Journal of Respiratory and Critical Care Medicine 2008, 177(12):1377-83.

5. Song J, Eichstaedt CA, Viales RR et al: Identification of genetic defects in pulmonary arterial hypertension by a new gene panel diagnostic tool. Clin Sci (Lond) 2016, 130(22):2043-52.

6. Rosenzweig EB, Morse JH, Knowles JA et al: Clinical Implications of Determining BMPR2 Mutation Status in a Large Cohort of Children and Adults With Pulmonary Arterial Hypertension. The Journal of Heart and Lung Transplantation 2008, 27(6):668-74.

7. Momose Y, Aimi Y, Hirayama T et al: De novo mutations in the BMPR2 gene in patients with heritable pulmonary arterial hypertension. Ann Hum Genet 2015, 79(2):85-91.

8. Machado RD, Southgate L, Eichstaedt CA et al: Pulmonary Arterial Hypertension: A Current Perspective on Established and Emerging Molecular Genetic Defects. Human mutation 2015, 36(12):1113-27.

9. Machado RD, Eickelberg O, Elliott CG et al: Genetics and genomics of pulmonary arterial hypertension. J Am Coll Cardiol 2009, 54(1 Suppl):S32-S42.

10. Liu D, Liu QQ, Eyries M et al: Molecular genetics and clinical features of Chinese idiopathic and heritable pulmonary arterial hypertension patients. Eur Respir J 2012, 39(3):597-603.

11. Lane KB, Machado, Rajiv D., Pauciulo MW et al: Heterozygous germline mutations in BMPR2, encoding a TGF-β receptor, cause familial primary pulmonary hypertension. Nat Genet 2000, 26:81-84.

12. Kabata H, Satoh T, Kataoka M et al: Bone morphogenetic protein receptor type 2 mutations, clinical phenotypes and outcomes of Japanese patients with sporadic or familial pulmonary hypertension. Respirology 2013:1076-82.

13. Thomson JR, Machado RD, Pauciulo MW et al: Sporadic primary pulmonary hypertension is associated with germline mutations of the gene encoding BMPR-II, a receptor member of the TGF-β family. J Med Genet 2000, 37:741–45.
